# Exploring the Free Energy Landscape: From Dynamics to Networks and Back

**DOI:** 10.1371/journal.pcbi.1000415

**Published:** 2009-06-26

**Authors:** Diego Prada-Gracia, Jesús Gómez-Gardeñes, Pablo Echenique, Fernando Falo

**Affiliations:** 1 Departamento de Física de la Materia Condensada, Universidad de Zaragoza, Zaragoza, Spain; 2 Instituto de Biocomputación y Física de Sistemas Complejos (BIFI), Universidad de Zaragoza, Zaragoza, Spain; 3 Departamento de Matemática Aplicada, ESCET, Universidad Rey Juan Carlos, Móstoles (Madrid), Spain; 4 Departamento de Física Teórica, Universidad de Zaragoza, Zaragoza, Spain; Stanford University, United States of America

## Abstract

Knowledge of the Free Energy Landscape topology is the essential key to understanding many biochemical processes. The determination of the conformers of a protein and their basins of attraction takes a central role for studying molecular isomerization reactions. In this work, we present a novel framework to unveil the features of a Free Energy Landscape answering questions such as how many meta-stable conformers there are, what the hierarchical relationship among them is, or what the structure and kinetics of the transition paths are. Exploring the landscape by molecular dynamics simulations, the microscopic data of the trajectory are encoded into a Conformational Markov Network. The structure of this graph reveals the regions of the conformational space corresponding to the basins of attraction. In addition, handling the Conformational Markov Network, relevant kinetic magnitudes as dwell times and rate constants, or hierarchical relationships among basins, completes the global picture of the landscape. We show the power of the analysis studying a toy model of a funnel-like potential and computing efficiently the conformers of a short peptide, dialanine, paving the way to a systematic study of the Free Energy Landscape in large peptides.

## Author Summary

A complete description of complex polymers, such as proteins, includes information about their structure and their dynamics. In particular it is of utmost importance to answer the following questions: What are the structural conformations possible? Is there any relevant hierarchy among these conformers? What are the transition paths between them? These and other questions can be addressed by analyzing in an efficient way the Free Energy Landscape of the system. With this knowledge, several problems about biomolecular reactions (such as enzymatic activity, protein folding, protein deposition diseases, etc.) can be tackled. In this article we show how to efficiently describe the Free Energy Landscape for small and large peptides. By mapping the trajectories of molecular dynamics simulations into a graph (the Conformational Markov Network) and unveiling its structural organization, we obtain a coarse grained description of the protein dynamics across the Free Energy Landscape in terms of the relevant kinetic magnitudes of the system. Therefore, we show the way to bridge the gap between the microscopic dynamics and the macroscopic kinetics by means of a mesoscopic description of the associated Conformational Markov Network. Along this path the compromise between the physical nature of the process and the magnitudes that characterize the network is carefully kept to assure the reliability of the results shown.

## Introduction

Polymers and, more specifically, proteins, show complex behavior at the cellular system level, *e.g.* in protein-protein interaction networks [1], and also at the individual level, where proteins show a large degree of multistability: a single protein can fold in different conformational states [2]–[4]. As a complex system [5],[6], the dynamics of a protein cannot be understood by studying its parts in isolation, instead, the system must be analyzed as a whole. Tools able to represent and handle the information of the entire picture of a complex system are thus necessary.

Complex network theory [7],[8] has proved to be a powerful tool used in seemingly different biologically-related fields such as the study of metabolic reactions, ecological and food webs, genetic regulatory systems and the study of protein dynamics [7]. In this latter context, diverse studies have analyzed the conformational space of polymers and proteins making use of network representations [9]–[12], where nodes account of polymer conformations. Additionally, some studies have tried to determine the common and general properties of these conformational networks [13],[14] looking at magnitudes such as clustering coefficient, cyclomatic number, connectivity, etc. Recently, trying to decompose the network in modules corresponding to the free energy basins, the use of community algorithms over these conformational networks have been proposed [15]. Although this approach has opened a promising path for the analysis of Free Energy Landscapes (FEL), the community based description of the network leads to multiple characterizations of the FEL and thus it is difficult to establish a clear map from the communities found to the basins of the FEL.

A similar approach, commonly used to analyze the complex dynamics, is the construction of Markovian models. Markovian state models let us treat the information of one or several trajectories of molecular dynamics (MD) as a set of conformations with certain transition probabilities among them [9],[16],[17]. Therefore, the time-continuous trajectory turns into a transition matrix, offering global observables as relaxation times and modes. In [16]–[18] the use of Markovian models is proposed with the aim of detecting FEL meta-stable states. However, the above approaches to analyze FELs of peptides involves extremely large computational cost: either general community algorithms or large transition matrices.

Finally, other strategies to characterize the FEL that have successfully helped to understand the physics of biopolymers, are based on the study of the Potential Energy Surface (PES) [3], [4], [19]–[21]. The classical transition-state theory [22] allows us to project the behavior of the system at certain temperature from the knowledge of the minima and transition states of the PES. This approach entails some feasible approximations, such as harmonic approximation to the PES, limit of high damping, assumption of high barriers, etc. These approximations could be avoided working directly from the MD data.

In this article we make a novel study of the FEL capturing its mesoscopic structure and hence characterizing conformational states and the transitions between them. Inspired by the approaches presented in [12],[15] and [16],[17], we translate a dynamical trajectory obtained by MD simulations into a Conformational Markov Network. We show how to efficiently handle the graph to obtain, through its topology, the main features of the landscape: conformers and their basins of attraction, dwell times, rate constants between the conformational states detected and a coarse-grained picture of the FEL. The framework is shown and validated analyzing a synthetic funnel-like potential. After this, the terminally blocked alanine peptide (Ace-Ala-Nme) is studied unveiling the main characteristics of its FEL.

## Methods

In this section we show the round way of the FEL analysis: the map of microscopic data of a MD into a Conformational Markov Network (CMN) and, by unveiling its mesoscopic structure, the description of the FEL structure in terms of macroscopic observables.

### Translating the FEL into a network

First, we encode a trajectory of a stochastic MD simulation into a network: the CMN. This map will allow us to use the tools introduced henceforth to analyze a specific dynamics of complex systems such as biopolymers.

#### Conformational Markov Network

The CMN has been proven to be a useful representation of large stochastic trajectories [10],[11],[15]. This coarse grained picture is usually constructed by discretizing the conformational space explored by the dynamical system and considering the hops between the different configurations as dictated by the MD simulation. In this way, the nodes of a CMN are the subsets of configurations defined by the conformational space discretization and the links between nodes account for the observed transitions between them. The information of the stochastic trajectory allows to assign probabilities for the occupation of a node and for the transitions between two different configurations. Defined as above, a CMN is thus a weighted and directed graph.

Our CMN is constructed as follows. The conformational space is divided into 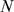 cells of equal volume, therefore every node 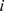 (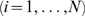) of the CMN contains the same number of possible configurations. Next, by evolving a stochastic trajectory enough time steps (of length 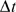) to assure the ergodicity condition we can define the final CMN set up. We assign to each node a weight, 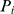, that accounts for the fraction of trajectory that the system has visited any of the configurations contained in node 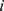 (the following normalization 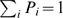 holds). Second, a value 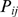 is assigned to each directional link, accounting for the number of hops from node 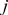 to node 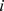. Note that transitions between configurations contained in the same node are also considered by 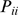, *i.e.* the network can also contain self-loops. Finally, the weights of the outgoing links from a node 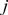, 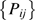, are conveniently normalized so that 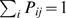.

The CMN constructed in this way, is described by a single matrix 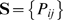 and a vector whose components are the occupation probabilities 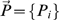. Hence, the matrix 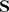 is the transition probability matrix of the following Markov chain,
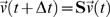
(1)where 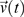 is the instant probability distribution of the system at time 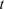. Since the matrix 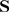 is ergodic and time invariant, one can compute the stationary distribution associated to the Markov chain, 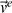, that satisfies 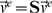. The latter stationary distribution has to be identical to the computed weights of the network nodes, 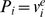 (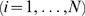), provided the stochastic trajectory is long enough. Moreover, the detailed balance condition,
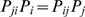
(2)must hold thus relating the elements of matrix 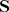 to the stationary probability distribution. Therefore, the transition matrix 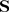 appears to be the minimal descriptor of the stochastic trajectory and, as consequence, of the CMN.

#### Markovity

Provided the MD trajectory is long enough to consider the sample in equilibrium, the weight-distribution of nodes in the CMN will be the stationary solution of Eq. (1) and detailed balance condition (2) will be fulfilled [23]. However, this property is not enough to consider the model Markovian: Although the continuous trajectory will be produced using Langevin dynamics (and therefore inherently Markovian in the phase space [24],[25]) the discrete representation of the CMN and the integration of momenta defies the Markovian character of our model [24], [26]–[28]. Several methods are proposed in the literature to validate Markov models [16],[27],[29]. In order to obtain a reliable description, specially about those magnitudes related to the time evolution of the system (see subsection Temporal hierarchy of basins), the time step 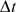 must be large enough to avoid memory effects [27].

A detailed check and discussion about the Markovian character of the networks shown in this article can be found in the Text S1.

#### Funnel-like potential

**Figure 1:**
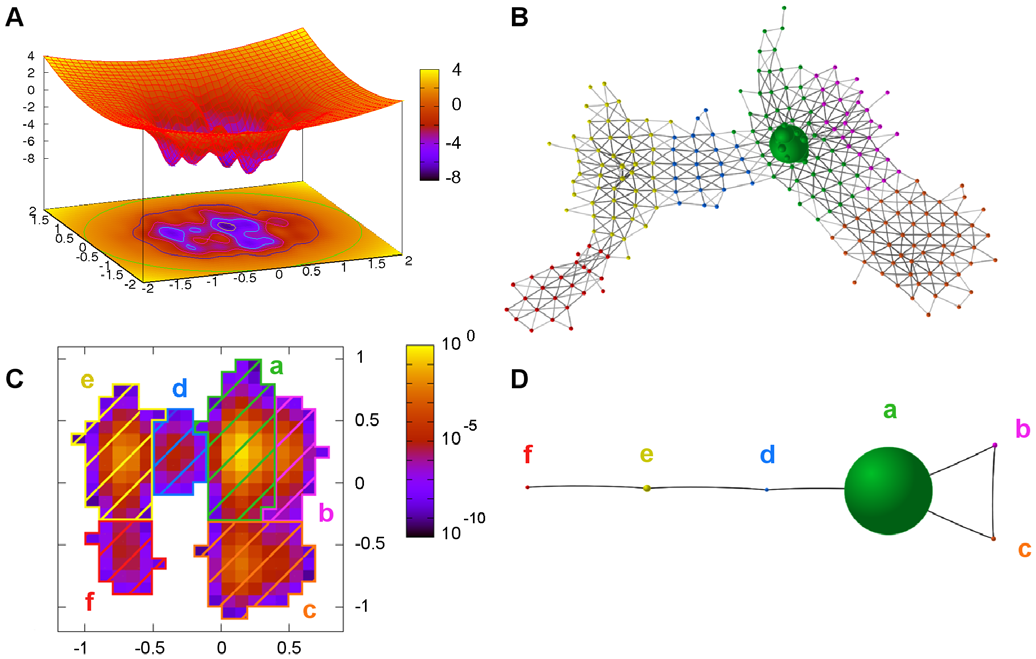
SSD algorithm applied to a synthetic funnel-like potential. (A) 2D funnel-like potential. (B) A stochastic trajectory is translated into a CMN where 6 sets of nodes (corresponding to different color) are the result of the SSD algorithm. (C) Recovering the spatial coordinates, the stationary probabilities of each node are shown in color code. The 6 basins detected are represented as color striped regions. (D) A coarse-grained CMN is built where new nodes take the role of the basins.

To illustrate the CMN approach and the methods presented below, we introduce here a synthetic potential energy function, that serve us as a toy model where results can be easily interpreted. This potential energy is reminiscent of that funnel surfaces recurrently found when the FEL of proteins are studied [30],[31]. In particular, we have considered a two-dimensional system where a particle moves in contact with a thermal reservoir and whose potential energy is given by,
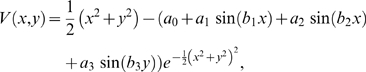
(3)where we have set 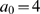, 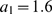, 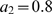, 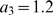, 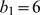, 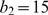 and 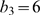. As shown in Figure 1A the above potential energy confines the movement of the stochastic trajectory inside a finite region of the conformational space. However, thermal fluctuations allow the particle to jump between several basins of attraction.

A stochastic trajectory has been simulated using an overdamped Langevin dynamics and the equations of motion have been integrated with a fourth order stochastic Runge-Kutta method [32]. Figure 1C shows the region of the conformational space visited by the particle. We have conveniently discretized the two-dimensional space into pixels of equal area and computed their corresponding occupation probabilities. Thereby, with the transition probabilities between pixels, the trajectory is represented as the CMN shown in Figure 1B. The question now is: can we recover the topology of the FEL (derived from Eq. (3)) from the CMN representation?

### Analyzing the FEL through the network

Up to now, we have illustrated the conversion of molecular dynamics data into a graph (the CMN). Now, we show how to efficiently obtain the thermo-statistical data from the mesoscopic description of the CMN.

#### Revealing structure: Conformational basins

Inspired by the deterministic steepest descent algorithm to locate minima in a potential energy surface we propose a *Stochastic Steepest Descent* (SSD) algorithm to define basins on the discretized FEL. Dealing with the nodes and links as we describe below, the proper structure of the CMN is unveiled to call the modules obtained conformational macro-states or basins.

Picking at random one node of the CMN, say 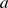, and an initial probability distribution 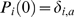 (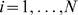), the Markov process relaxes according to 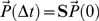. The whole probability concentrated in node 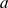 at time 0, in a single time step 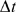, evolves driving the maximum amount of probability down hill over the FEL. The next node 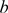 in the descendent pathway from 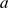 is taken by following the link that carries maximum probability flux. Focusing again all the probability in node 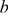, 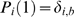 we continue the pathway from 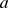 towards a local FEL minimum by identifying the next node 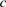 for which the probability current 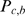 is maximal. Iterating this operation for each node of the CMN, we obtain a set of disconnected descent pathways that help us to define the basins of attraction.

We establish formally the above procedure assisted by a vector 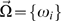 (with 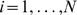) that label the nodes:

1. We start by assigning 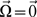.
2. Select at random a node 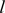 with 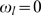 (*i.e.*, 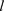 has not been labeled yet) and start to write an auxiliary list 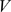 of nodes adding 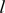 as the first entry in this list.
3. Search, within the neighbors of the node 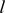, a node 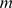 so that 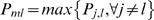 (If the max value of *P_j,l_* is reached for several neighbors of *l* (degeneracy), we choose at random one of them.) and check which of the following options is fulfilled:
  A If 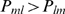 and 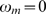: add 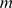 to the list 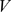 and go again to *(iii)* taking 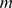 in the place of 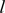.
  B If 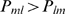 and 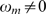 then write the labels of all the nodes in the list 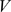 as 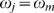 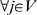. The process continues going to step *(ii)*.
  C If 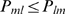 the link 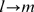 is removed from the graph. The process returns to step *(iii)* with the next exception: since this step has been iterated 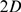 times for the same node 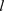 (being 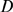 the number of coordinates discretized to construct the CMN), 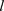 is stated as local minimum and 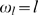. In this case 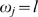 for those nodes 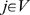 and the process comes back to step *(ii)*.

The whole procedure ends when no nodes unlabeled remain in the CMN, 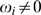 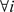. The restriction introduced in step (*iii*.C) with the dimensionality 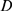 avoids a transition from a local minimal energy configuration to any other node of the same basin or to a deeper local minimum of a different basin. When every node of the CMN has been visited, the conformational space is completely characterized and we have thus traced all the maximum descent pathways from any node to the local FEL minima. Finally, all those nodes with the same label 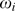 belong to the same FEL basin and therefore they are associated to the same conformational state. The result of the procedure is the partition of the CMN in a set of modules which correspond to basins of attraction of the discretized conformational space.

To illustrate the basin decomposition of a CMN, the SSD algorithm has been applied to the funnel-like potential. The result is the detection of six basins in agreement with the number of local minima in its FEL (Figure 1B and 1C).

#### Comparing with other community algorithms

With the aim of studying biomolecules and systems with high degree of dimensionality, the way to detect these FEL basins must be computationally efficient. The method described above takes a computational time 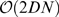, once the 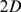 largest hooping probabilities 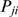 are computed for all the nodes in the network. Additionally, the method is deterministic providing with a unique partition of the CMN into different modules. These two characteristics make this analysis faster and more straightforward than any other partitioning method [33]. These advantages come from the knowledge of the physical meaning of links and nodes of CMNs. In Text S2 other community algorithms (Newman's modularity and Markov Clustering algorithm) are tested over our toy model system. None of the algorithms reported in the Text S2 give a satisfactory result mapping the modules obtained with the free energy basins.

#### Coarse-grained CMN

To get a more comprehensible representation of the FEL studied, a new CMN network can be built by taking the basins as nodes. The occupation probabilities of these nodes as well as the transition probabilities among them can be obtained from those of the original CMN as
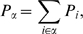
(4)

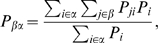
(5)where 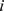 and 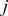 are indexes relative to the nodes of the original CMN and 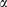 and 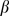 are indexes for the basins (new nodes). Note that the new coarse-grained CMN has its weights normalized and fulfills the detailed balance condition Eq. (2). Figure 1D shows the corresponding coarse-grained for the funnel-like potential.

The weighted nodes and links have a clear physical meaning [19]. Considering the transition state 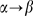 and assuming local “intra-basin” equilibrium, the rate constant of this transition is 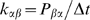 (where 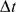 is the time interval between snapshots used to make the original CMN). The relative free energy of the basin 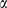, taking basin 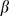 as reference, is 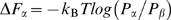. Besides, the expected waiting time to escape from 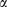 to any adjacent basin is 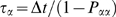 [34]. Other magnitudes, such as first-passage time for inter-basins transitions and other rate constants relaxing the local equilibrium condition [19],[34] can also be computed from the original CMN.

The ability to define the proper regions of the conformational space in an efficient way let us compute physical magnitudes of relevance. For instance, the coarse-grained CMN is nothing but a graphical representation of a kinetic model with 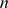 (the number of basins) coupled differential equations:
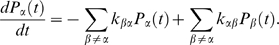
(6)


#### Free Energy hierarchical basin organization

The first hierarchy aims to answer the following question: What is the structure of the CMN when nodes with lower weight than a certain threshold are removed together with their links? Let us take the control parameter 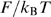 as the threshold to restrict the existence of the nodes in the original CMN. Where 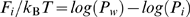 is the “*adimensional free energy*” of a node 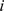 relative to the most weighted node 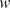.

With the above definitions we start a CMN reconstruction by smoothly increasing the threshold from its zero value. At each step of this process, we obtain a network composed of those nodes with free energy lower than the current threshold value. As the free-energy cut-off increases, new nodes emerge together with their links. These new nodes may be attached to any of the nodes already present in the network or they can emerge as a disconnected component. At a certain value of 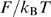, some components of the network become connected by the links of a new node incorporated at this step. A Hoshen-Kopelman like algorithm [35] is used to detect the disconnected components of the network at each value of the threshold used: from zero until all the 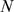 nodes of the CMN were already attached.

**Figure 2:**
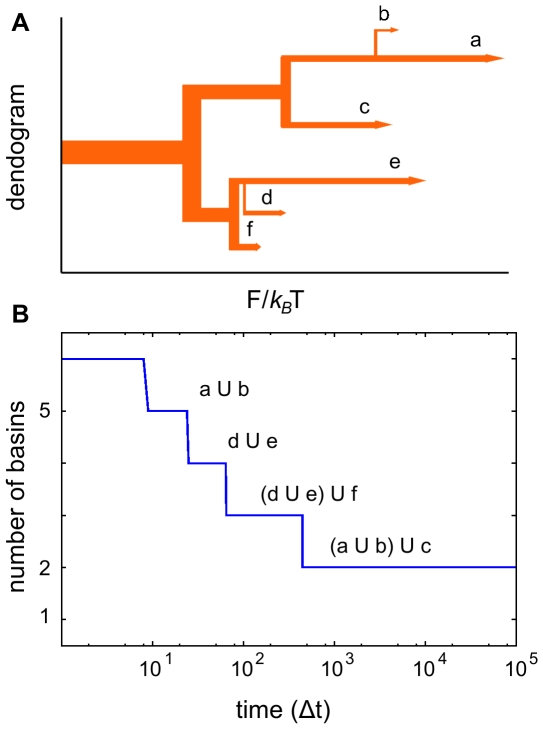
Hierarchies of the basins detected for the funnel-like potential. (A) Free Energy hierarchy: based on the relative free-energy of the nodes. (B) Temporal hierarchy: number of basins defined by SSD for the different networks built by Eq. (7). The original basins merge in function of time. Both hierarchies reveals a coarse-grained behavior of two macro-states: 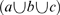 and 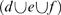.

This bottom-up network reconstruction provides us with a hierarchical emergence of nodes along with the way they join together. This picture can be better described by a process of basins emergence and linking that is easily represented by means of a basin dendogram. This representation let us guess at first glance the hierarchical relationship of the conformational macro-states and the height of the barriers between them. Let us remark that the transition times cannot be deduced from these qualitative barriers since the entropic contribution or the volume of the basin are not reflected in this diagram. The basins family-tree obtained for the funnel-like (see Figure 2A) reveals that, despite of having a two-dimensional potential with the shape of a funnel, one cannot describe it as a sequence of metastable conformations that drive the system to the global minimum. Moreover, the diagram shows a roughly similar behavior as for a double asymmetric well, composed by two sets of basins: 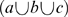 and 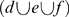.

#### Temporal hierarchy of basins

The CMN representation of a MD simulation provides with another hierarchical relationship that is meaningful to understand the behavior of the biological systems. The links of the original CMN have been weighted according to the stochastic matrix 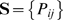. Taking into account the Markovian character of the process, we can make use of the Chapman-Kolmogorov equation to generate new transition matrices at times 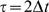, 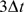, etc… Formally, the Markov chain at sampling time 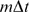 is defined by the matrix:
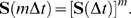
(7)


For each value of 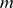 a new CMN is defined. This family of CMNs have different weighted links but the same weights 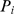 for the nodes as the original one (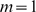). It is worth to discuss the behavior of the matrix 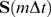. In the limit 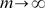 we have 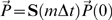 independently of the initial state 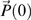. This means that any node is connected to a given node of the network with the same weight, regardless of the initial source. Therefore, only one basin would be detected by the SSD algorithm since every node is connected with the most weighted link to the most weighted node in only one step.

From the original 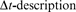 of the FEL to the integrated (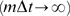) one, we can devise another algorithm to establish a second hierarchy of the basins by performing the next two operations: First, for each value of 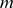 a new CMN is generated by constructing the matrix 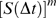 and second, the SSD algorithm is applied to this new CMN. The process finishes when only a basin (the whole network) is detected (for large enough values of 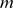). By using this technique we can observe how basins merge with others at different time scales (labeled by the integer 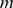).

The result of this procedure performed for the funnel-like potential is shown in Figure 2B. At 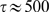 only two basins are observed: 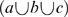 and 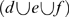, being the largest plateau observed for any of the nontrivial arrangement of basins found. Therefore, the macroscopic description in time is in agreement with the Free Energy hierarchy described previously. It is clear that the number of basins decrease as 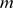 increases. One should be aware that the concept of basin depends dramatically on the time resolution at which the CMN is built, and this time limits also the resolution in the FEL structure. Note also that this procedure provides with useful information similar to the *structural decorrelation time* [36].

## Results

### The alanine dipeptide

**Figure 3:**
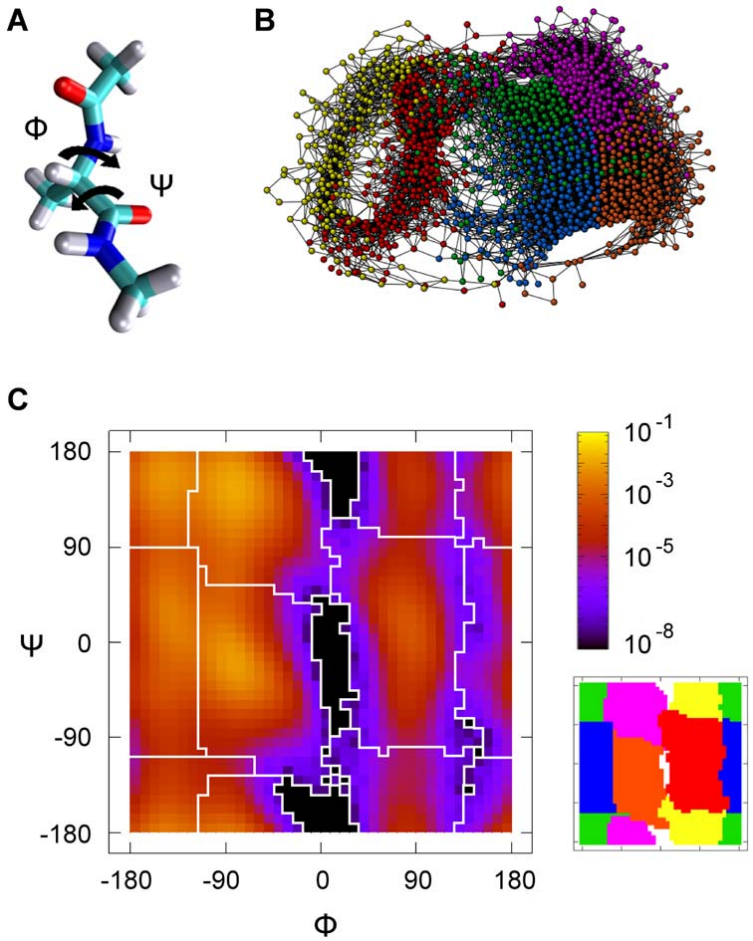
Free energy basins of the Alanine dipeptide. (A) The dialanine dipeptide with the angles 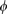 and 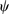. (B) Plot of the CMN generated. The 6 sets of nodes (corresponding to different colors) are the result of the SSD algorithm. (C) Left: Ramachandran plot with the probability of occupation of the cells used to build the CMN. The boundaries of the free energy basins are shown in white. Right: the 6 basins represented as regions of different color. (Color code: 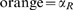, 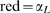, 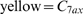, 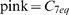, 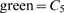 and 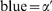).

The alanine dipeptide, or terminally blocked alanine peptide (Ace-Ala-Nme, Figure 3A), is the most simple “biological molecule” that exhibits the common features shown by larger biomolecules. Despite of its simplicity, this system has more than one long-life conformational state with different transition pathways. Since the first attempt by Rossky and Karplus [37] to model this dipeptide solvated, this system has been widely studied in theoretical works [38]–[41]. The alanine dipeptide has been also the appropriate molecule to test tools to explore the FEL [15],[16],[42] and, specifically, to study reaction coordinates [41],[43].

The alanine dipeptide has two slow degrees of freedom, the rotatable bonds 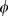 (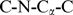) and 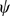 (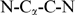) (see Figure 3A). The FEL projected onto these dihedral angles let us identify the conformational states that characterize the geometry of biopolymers, namely: alpha helix right-handed (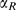), alpha helix left-handed (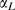), beta strands (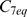, 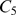), etc. The number of local minima in the (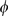, 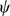) space depends on the effective potential model used to simulate the system. Up to date, electronic structure calculations have identified a total of nine different conformers [44]. Regarding MD simulations different conformational states have been observed: *(i)* using classical force fields with explicit solvent up to six conformers are detected [16],[38],[39], *(ii)* at least four stable states by using implicit solvent [15],[38],[40], and *(iii)* two stable conformers in vacuum conditions [38],[41]. On the other hand, since the angles 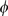 and 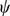 seem appropriate to distinguish the metastable states, the kinetics between them is not accurately described with this choice of reaction coordinates, the solvent coordinates and/or other internal degrees of freedom must be taken into account [41],[43].

We have used *SSD* algorithm to detect the local minima and their corresponding basins for this molecule in the 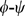 space. For this purpose, a Langevin MD simulation of 250 ns has been performed at a temperature of 400 K (see Text S3 for further details). Additionally the CMN has been built dividing the 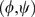 Ramachandran plot into cells of surface 9°×9° (40×40) and taking dialanine conformations at time intervals of 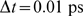. The resulting CMN have a total of 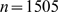 nodes and 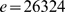 directed links.

The 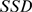 algorithm applied to the CMN network reveals 6 basins. Figure 3B shows the resulting network where nodes belonging to the same basin take the same color. Bringing back this information to the Ramachandran map, these 6 sets of nodes define 6 regions represented in Figure 3C. To better illustrate this division, other representation, where each region has a different color, is shown. By comparing with previous studies on this molecule, we identify the regions in orange, red, yellow and pink with conformers 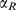, 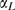, 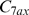 and 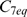 respectively [38],[45]. Besides, region green corresponds to conformer 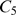 and the blue one to 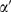 [16],[45]. Remarkably, the basin 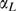 (one of the less populated state) has been visited 1155 times with a mean stay time of 4.20 ps.

We now look at the coarse-grained picture of the FEL by describing the properties of the 6 basins detected. The different weights of the basins are related to the free energy of the corresponding conformational macro-states. In Table 1 these energy differences 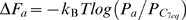 are shown, taking the most populated basin as the energy reference 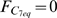. The lowest free energy basins correspond to configurations with *φ*≤0° (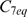, 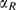, 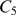 and 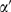), whereas the two other conformers located in the region *φ*≥0° have the highest free energy but the largest dwell time. Moreover, we have also analyzed the trapping efficiency of each basin by reporting the mean escape time (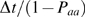) as well in Table 1.

**Table 1:** Relative free energies and Mean Escape Time of the basins defined by SSD.

Basin	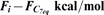	Mean Escape Time (ps)
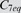	0.00	0.52
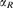	0.45	0.42
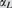	2.42	4.20
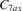	3.84	0.71
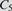	0.55	0.28
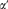	0.90	0.23

**Figure 4:**
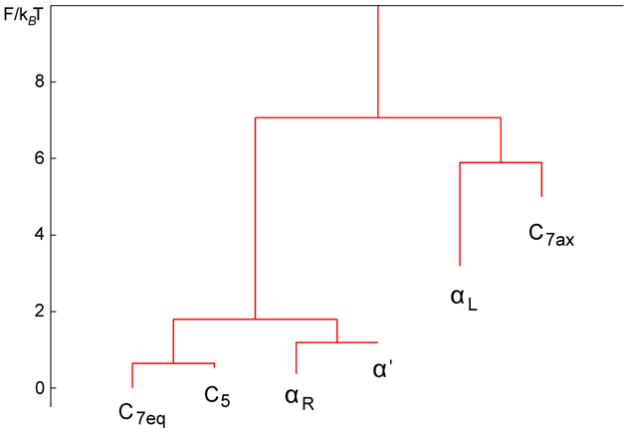
Dendogram based on the relative Free Energy of the CMN nodes. Two sets of basins are clearly distinguished with a high free energy barrier in between: (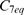, 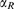, 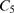, 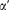) and (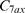, 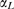). Note that 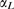 looks like the conformer with the largest dwell time, in agreement with data in Table 1.

The FEL can be represented as a dendogram, see Figure 4, where the hierarchical map of the conformers based on Free Energy gives at first glance a global picture of the landscape. Remarkably, the conformer 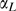, despite of having one of the highest free energy, looks like the metastable state with longest life. This result is supported by the values of Mean Escape Time shown in Table 1.

The alanine dipeptide has been also studied because of its “fast” isomerization 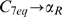 and a “slow” transition 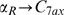. Our coarse-grained picture of the FEL also allows us to extract information about these transitions. In Table 2 we show some of the relevant characteristic transition times from a basin *a* to an adjacent basin *b*, 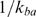. [The whole data is shown in the Text S3.] Transitions between basins with the same sign of 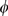 are remarkably faster (*e.g* 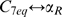 and 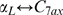). While slow transitions are observed for those hops crossing the line *φ* = 0° (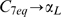 and 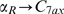), showing them as rare events. Instead, the alanine dipeptide finds more easily paths to go to *φ*≥0° conformers through 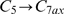 and 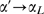 by crossing *φ* = 180°.

**Table 2:** Characteristic times for direct inter-basins transitions.

*a→b*	*1/K*_*ba*_ (ps)		
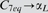	1968.34	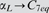	88.24
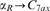	58011.74	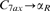	815.87
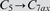	393.75	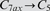	6.63
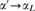	400.57	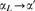	58.47
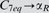	3.32	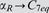	1.88
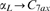	4.80	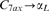	0.78

**Figure 5:**
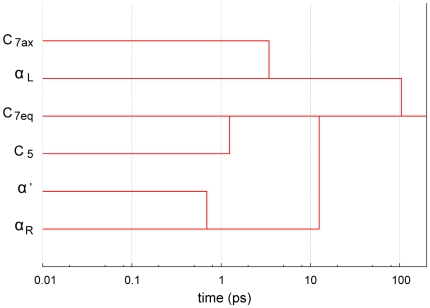
Dendogram based on the temporal hierarchy of basins. In around 100 ps the peptide finds the way to reach the global minimum, conformer 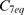, from any basin.

To round off the description of the FEL, the dendogram corresponding to the temporal hierarchy is shown in Figure 5. From the figure, it becomes clear that the behavior of the dialanine depends on the time scale used for its observation. Again, the same two different sets of conformers are distinguished from this hierarchy. Additionally, the global minimum conformer is reached in around 100 ps from any basin.

Finally, the magnitudes computed here for the alanine dipeptide would allow to construct a first-order kinetic model of 6 coupled differential equations as Eq. (6) (assuming equilibrium intra-basin). This model contains the same information as the kinetic model by Chekmarev et al. for the irreversible transfer of population from 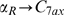 [40].

## Discussion

Hierarchical landscapes characterize the dynamical behavior of proteins, which in turn depends on the relation between the topology of the basins, their transitions paths and the kinetics over energy barriers. The CMN analysis of trajectories generated by MD simulations is a powerful tool to explore complex FELs.

In this article, we have proposed how to deal with a CMN to unveil the structure of the FEL in a straightforward way and with a remarkable efficiency. The analysis presented here is based on the physical concept of basin of attraction, making possible the study of the conformational structure of peptides and the complete characterization of its kinetics. Note that this has been done without the estimation of the volume of each conformational macro-state in the coordinates space and without the ‘a priori’ knowledge of the saddle points or the transition paths from a local minimum to another.

On the other hand, the framework introduced in the article provides us with a quantitative description of the dialanine's FEL, coming up directly from a MD dynamics at certain temperature. The peptide explores its landscape building the corresponding CMN and the success of extracting the relevant information is up to the ability of dealing with it. Neither the FE basins were defined by the unique criterion of clustering conformations with a geometrical distance [46], nor the rate constants were projected from the potential energy surface [19],[20]. Moreover, the conformers and their properties were computed from the MD with the only limitation of the discretization of time and space.

Although we have applied the method to low dimensional landscapes, we expect that high dimensional systems could be also studied, by the combination of this technique with the usual methods to reduce the effective degrees of freedom (like principal component analysis or essential dynamics). In conclusion, the large amount of information obtained by working with the CMN, its potential application to any peptide with a large number of monomers, and the possibility of performing the analysis on top of CMN constructed via several short MD simulations [47], make the approach presented here a promising way to describe the FEL of a protein.

## Supporting Information

Text S1 Checking Markovity.
(0.56 MB ZIP)

Text S2 Comparing with community algorithms.
(0.18 MB ZIP)

Text S3 More on Alanine dipeptide.
(<0.01 MB ZIP)
