# Supplementary figures and images for: Exploring the Free Energy Landscape: From Dynamics to Networks and Back
Source: PLoS Comput Biol. 2009 Jun 26;5(6):e1000415. doi: 10.1371/journal.pcbi.1000415 (PMC2694367; doi:10.1371/journal.pcbi.1000415)

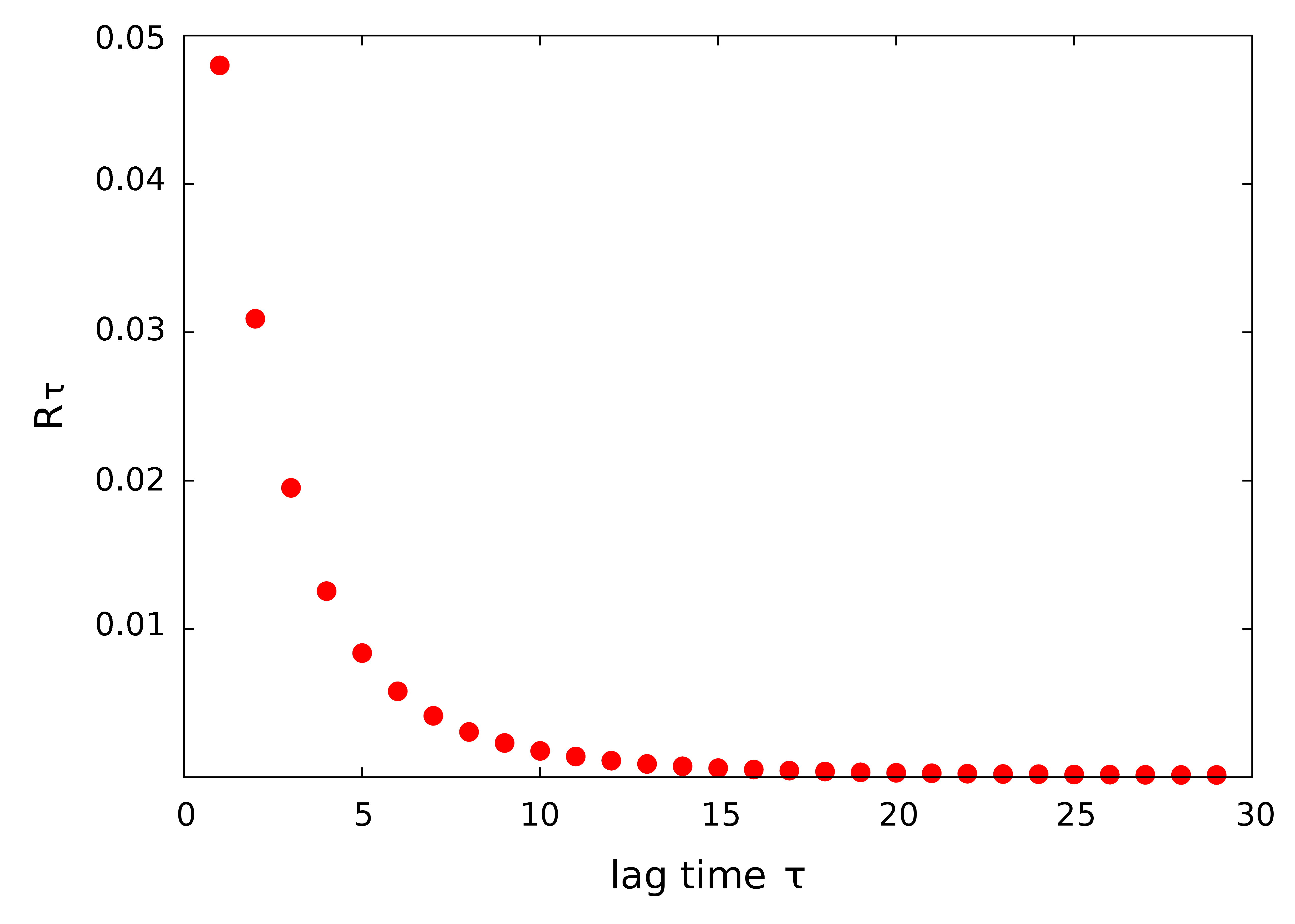

Supplement: Text S1 — Checking Markovity. (0.56 MB ZIP) [file pcbi.1000415.s001.zip › FigureS1.tif]

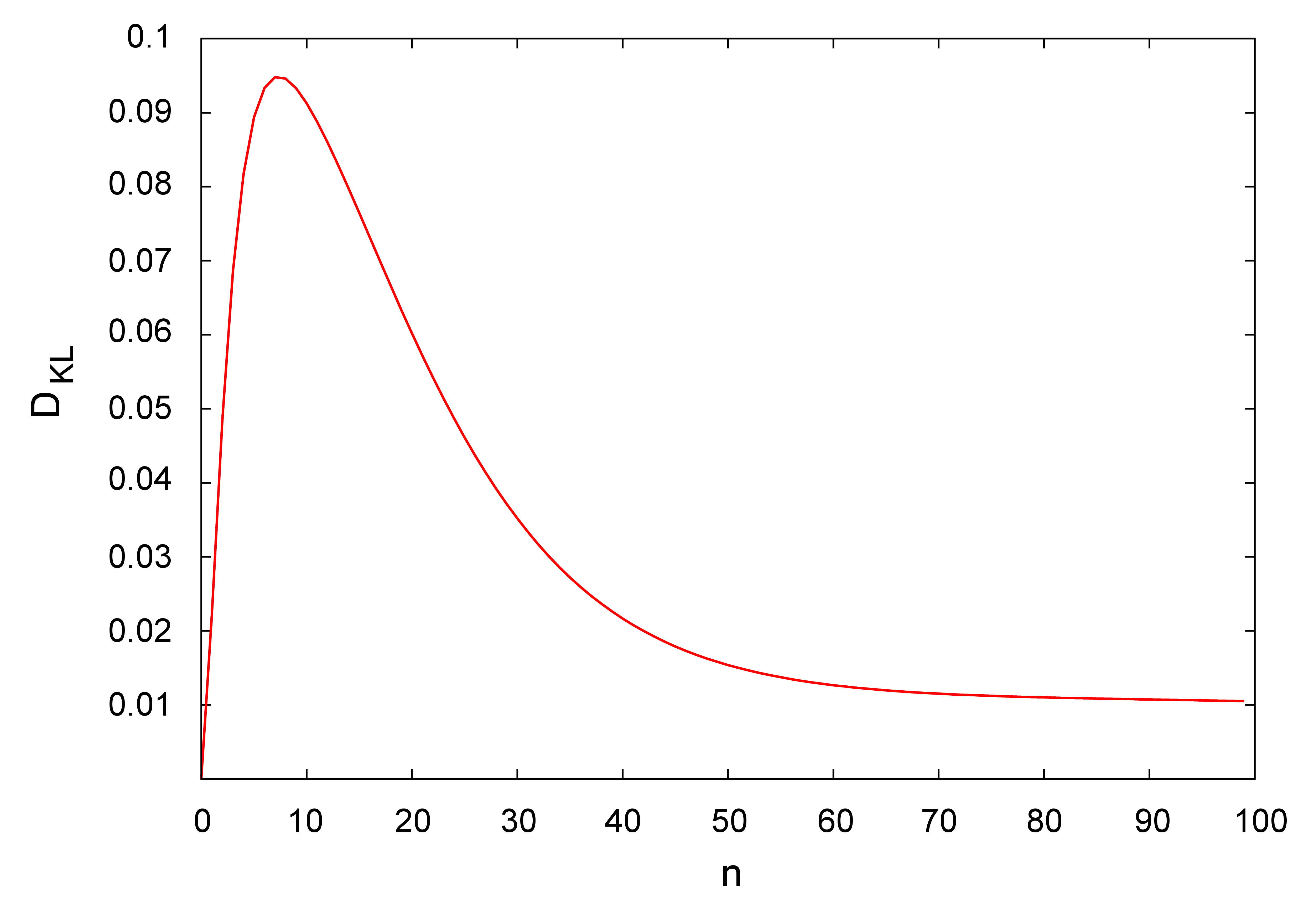

Supplement: Text S1 — Checking Markovity. (0.56 MB ZIP) [file pcbi.1000415.s001.zip › FigureS2.tif]

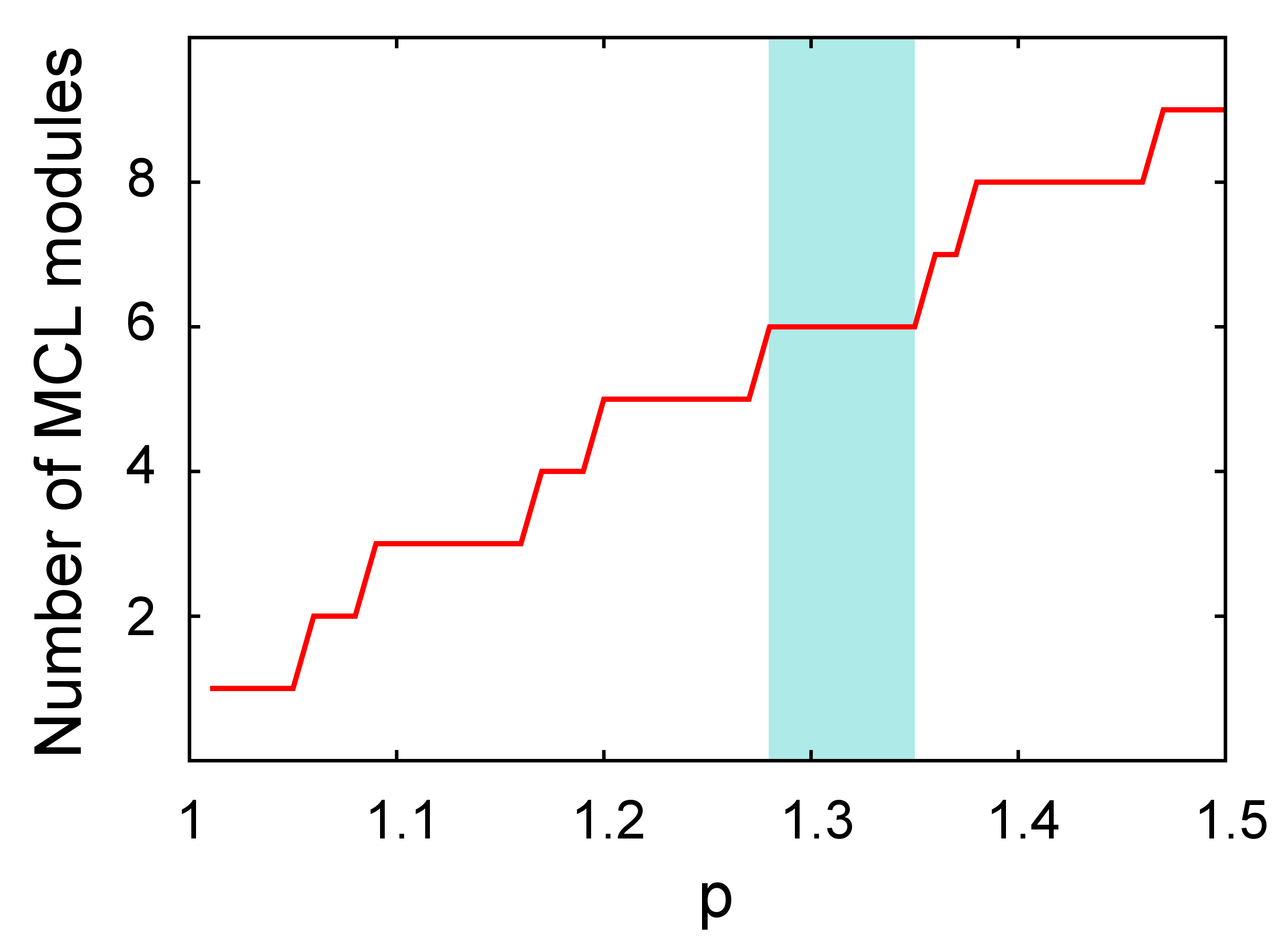

Supplement: Text S2 — Comparing with community algorithms. (0.18 MB ZIP) [file pcbi.1000415.s002.zip › FigureS3.tif]
